# Mutational analysis of two residues in the DYRK homology box of the protein kinase DYRK1A

**DOI:** 10.1186/s13104-018-3416-4

**Published:** 2018-05-15

**Authors:** Esti Wahyu Widowati, Simone Bamberg-Lemper, Walter Becker

**Affiliations:** 1 Institute of Pharmacology and Toxicology, RWTH Aachen University, Aachen, Germany; 2 Chemistry Study Program, Faculty of Science and Technology, State Islamic University (UIN) Sunan Kalijaga, Yogyakarta, Indonesia

**Keywords:** DYRK1A, DH box, Protein kinase, Tyrosine autophosphorylation, In vitro translation

## Abstract

**Objective:**

Dual specificity tyrosine phosphorylation-regulated kinases (DYRK) contain a characteristic sequence motif (DYRK homology box, DH box) that is located N-terminal of the catalytic domain and supports the autophosphorylation of a conserved tyrosine during maturation of the catalytic domain. Two missense mutations in the DH box of human DYRK1B were recently identified as causative of a rare familiar form of metabolic syndrome. We have recently shown that these amino acid exchanges impair maturation of the kinase domain. Here we report the characterization of DYRK1A point mutants (D138P, K150C) that correspond to the pathogenic DYRK1B variants (H90P, R102C).

**Results:**

When expressed in HeLa cells, DYRK1A-D138P and K150C showed no significant difference from wild type DYRK1A regarding the activating tyrosine autophosphorylation or catalytic activity towards exogenous substrates. However, both DYRK1A variants were underphosphorylated on tyrosine when expressed in a bacterial cell free in vitro translation system. These results suggest that D138 and K150 participate in the maturation of the catalytic domain of DYRK1A albeit the mutation of these residues is compensated under physiological conditions.

**Electronic supplementary material:**

The online version of this article (10.1186/s13104-018-3416-4) contains supplementary material, which is available to authorized users.

## Introduction

Dual-specificity tyrosine phosphorylation-regulated kinases (DYRK) constitute a family of protein kinases that acquire the mature, active conformation of the catalytic domain by tyrosine autophosphorylation. This reaction takes place as a one-time event during or immediately after translation [1, 2]. N-terminal to the catalytic domain, DYRKs harbor a conserved sequence motif termed DH-box (DYRK homology box [3]) that was shown to be critical for tyrosine autophosphorylation of *Drosophila* dDYRK2 [4].

Two missense variants of the human *DYRK1B* gene were recently found to be associated with a familial form of metabolic syndrome [5]. Both substitutions (H90P and R102C) affect residues in the DH-box that are not conserved among DYRKs [6]. In a previous study, we have investigated the molecular effects of the pathogenic mutations. Both substitutions impaired tyrosine autophosphorylation in mammalian cells and resulted in the aggregation of underphosphorylated DYRK1B [6]. Nevertheless, a significant portion of DYRK1B-H90P and DYRK1B-R102C was able to mature and showed normal kinase activity when isolated from the soluble fraction.

DYRK1A is a paralog of DYRK1B that shares 85% sequence identity with DYRK1B in the catalytic domain and the neighboring DH box. It is the best characterized member of the DYRK family regarding its biochemical properties and the mechanism of maturation [2, 7, 8]. The crystal structure of autophosphorylated DYRK1A revealed extensive interactions between amino acids in the DH box and the catalytic domain, suggesting that the DH box contributes to the conformational stability of the domain fold [8]. However, the DYRK1A residues corresponding to those that are mutated in the pathogenic DYRK1B variants are exposed to the surface of the protein and are not engaged in interactions with the catalytic domain.

Stimulated by our characterization of the pathogenic DYRK1B missense variants, we have also investigated whether the corresponding DH box mutations of DYRK1A impair the tyrosine autophosphorylation in the activation loop that is linked to the maturation of catalytic domain.

## Main text

### Results and discussion

**Fig. 1:**
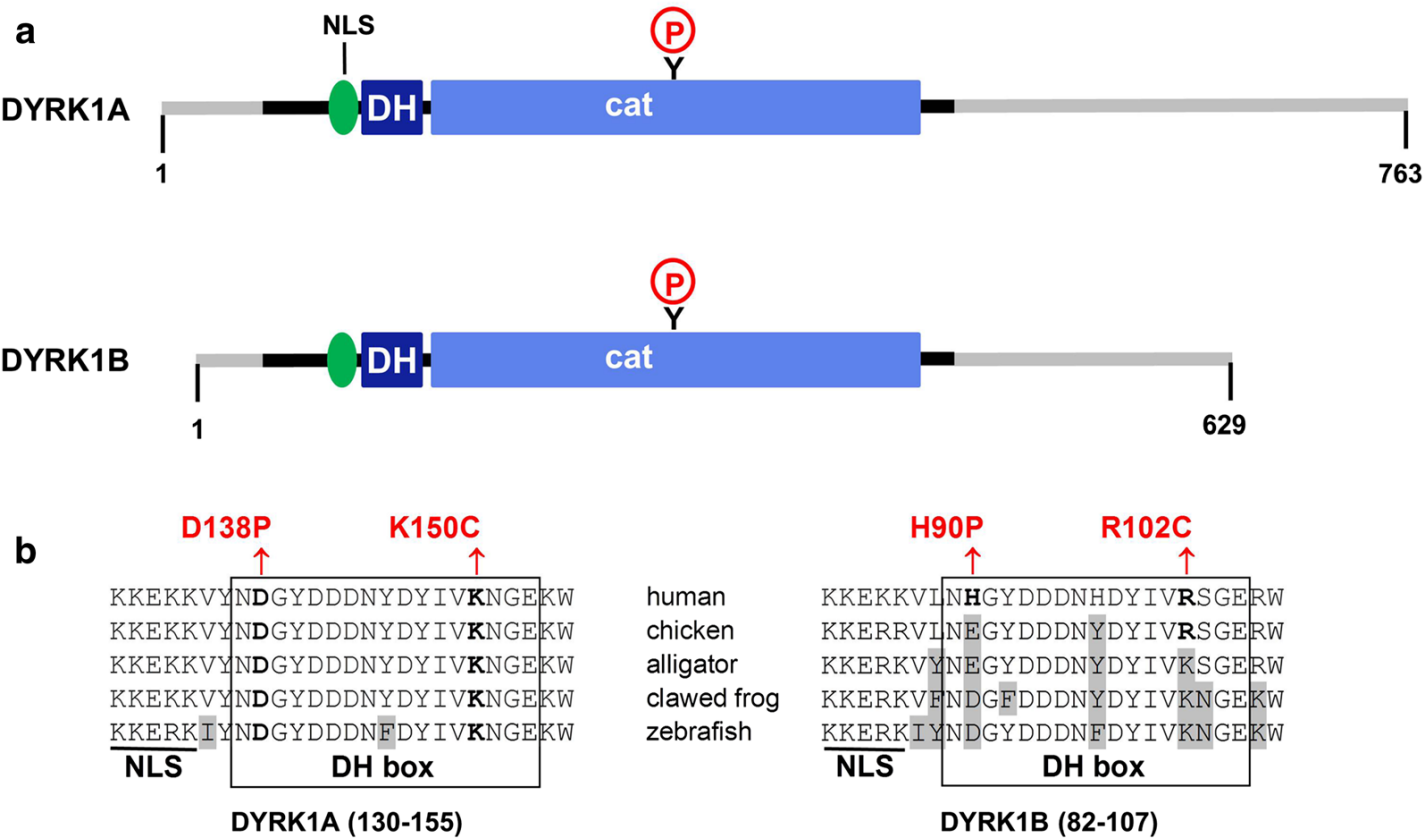
Structure of DYRK1A. **a** Domain structure DYRK1A and DYRK1B. The DH box is localized between the nuclear localization signal (NLS) and the catalytic domain (cat). The circled P (red) indicates the autophosphorylation of the tyrosine (Y) in the activation loop. Non-conserved N- and C-terminal region are shown in grey. **b** Sequence conservation of the DH box in DYRK1A and DYRK1B. The alignments illustrate that H90 and R102 in human DYRK1B are not conserved in vertebrates. The corresponding positions in DYRK1A are occupied by D138 and K150, which are conserved in evolution. Amino acids deviating from the human DYRK1A or DYRK1B sequences are shaded. The pathogenic variants in DYRK1B and the corresponding substitutions in DYRK1A are shown in red

The DH box is located directly adjacent to the catalytic domain of DYRKs and shows high sequence similarity between DYRK1A and DYRK1B (Fig. 1a, b). However, the amino acids affected by the pathogenic missense mutations in DYRK1B (H90 and R102) are not conserved in non-mammalian vertebrates (Fig. 1b). In contrast, the corresponding residues in DYRK1A are invariable, suggesting that the mutation of these residues might have more severe consequences in DYRK1A than observed in DYRK1B.

**Fig. 2:**
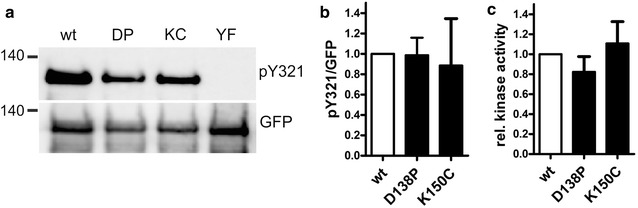
Effect of the mutations in HeLa cells. **a**, **b** Autophosphorylation on Tyr321. Wild type (wt) and mutant GFP-DYRK1A constructs (DP and KC) were immunoprecipitated from transiently transfected HeLa cells. Phosphorylation of Y321 in the activation loop was determined by immunoblot analysis. **a** Illustrates the Western blots of a representative experiment. DYRK1A-Y321F (YF) was included as a control for antibody specificity. The column diagram **b** shows the results (means and SD) of six independent experiments. Relative tyrosine phosphorylation was calculated by relating pY321 band intensities to total protein immunoreactivity. Results are presented relative to WT. **c** Catalytic activity. Immunoprecipitates were subjected to radiometric kinase assays with the peptide substrate DYRKtide. *In vitro* kinase activities were normalized to the amount of the respective GFP-DYRK1A fusion protein as quantified by immunoblot analysis. Pairwise differences between WT and mutant DYRK1A in four independent experiments were not significant (One sample t test, p > 0.05)

To mimic the deleterious DYRK1B variants in the DH box of DYRK1A, we replaced the respective amino acids in DYRK1A by those that are encoded by the mutant *DYRK1B* alleles in the human patients. First, we examined whether these substitutions (DYRK1A-D138P and DYRK1A-K150C) affected tyrosine autophosphorylation of DYRK1A, which serves as a marker for successful maturation and correct folding of the kinase domain. Wild type and mutant GFP-DYRK1A constructs were immunoprecipitated from transiently transfected HeLa cells to determine the phosphorylation status of Y321 in the activation loop (Fig. 2a). No difference between wild type and mutant DYRK1A was observed under these conditions. We have recently found that a pathogenic DYRK1A variant (L295F) with normal content of phosphotyrosine exhibited reduced catalytic activity in kinase assays [9]. Therefore, we measured the enzymatic activity of DYRK1A-D138P and K150C by immunocomplex kinase assays. Neither of the DYRK1A variants differed significantly in their catalytic activity towards an exogenous peptide substrate (Fig. 2c). In the analogous assay, we had also observed no significant effect of the respective amino acid exchanges on DYRK1B catalytic activity and only a small reduction of tyrosine phosphorylation in DYRK1B-H90P [6]. In aggregate, these results show that D138 and K150 are not essential for the folding and maturation of the catalytic domain in DYRK1A in HeLa cells.

**Fig. 3:**
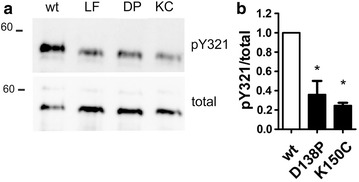
Autophosphorylation of DYRK1A point mutants in vitro. Wild type and mutant DYRK1A constructs were expressed in vitro by coupled transcription-translation (see ``Materials and methods''). Samples were incubated for 90 min at 37 °C before tyrosine autophosphorylation of the reaction products was assessed by immunoblot analysis **a**. DYRK1A-L295F was included as a control because this mutation is known to reduce tyrosine autophosphorylation in this assay [9] For quantification, pY321 signals were normalized to the total amounts of recombinant DYRK1A (**b**) (means and s.d., n = 3). The differences in relative tyrosine autophosphorylation between wild type and mutant were tested for statistical significance by One sample t test (*p < 0.05)

DYRK1A interacts with the HSP90/CDC37 chaperone system [10]. We employed an in vitro-translation system that is reconstituted from recombinant proteins and purified *E. coli* ribosomes to examine the effect of the DH box mutations in the absence of any chaperones or other proteins that might be involved in DYRK1A maturation. With the help of this approach, we have recently shown impaired tyrosine autophosphorylation of DYRK1A-L295F in vitro, although phosphotyrosine levels were normal in mammalian cells [9]. For this experiment, we used a DYRK1A deletion (DYRK1A_28–499_) construct which lacked the C-terminal sequence regions that are subject to truncation in *E. coli*. The C-terminal domain of DYRKs is not evolutionary conserved and, in contrast to the N-terminal region ([7, 11–13]), does not seem to be implicated in the folding and maturation of the kinase domain. Under these conditions, the DH box mutants, D138P and K150C were significantly underphosphorylated on Y321 relative to wild type DYRK1A (Fig. 3).

### Concluding remarks

Taken together, these results show that the highly conserved D138 and K150 in the DH box are not indispensable for the enzymatic function of DYRK1A in mammalian cells. However, both mutations impaired the co-translational tyrosine autophosphorylation when DYRK1A was expressed by in vitro-translation. Thus, cell-free expression revealed a minor impairment of the maturation process that seems to be compensated in a cellular environment, possibly by the action of chaperones or other potential co-factors. Alternatively, the absence of the C-terminal domain in the bacterial expression constructs may have uncovered an enhanced vulnerability of the maturation process in the point mutants. In either of these scenarios, our result is consistent with the hypothesis that the DH box has a function in the activation of DYRKs by tyrosine autophosphorylation in the activation loop [1, 2, 4].

### Materials and methods

#### Plasmids

The plasmid for mammalian expression of GFP-DYRK1A has been described previously [12]. Point mutants of DYRK1A were generated using the QuikChange method. All mutagenesis plasmids were verified by DNA sequencing. The construction of the vector for bacterial in vitro-translation (pET-ST2-DYRK1A_28–499_) is documented in Additional file 1.

#### Antibodies

Goat polyclonal antibodies were used to detect GFP (Rockland Immunochemicals (#600-401-215; RRID:AB_828167) or DYRK1A (Everest Biotech, #EB11483). A polyclonal rabbit antibody originally developed against pTyr361 in HIPK2 (Thermo Fischer Scientific, #PA5-13045; RRID:AB_10987115) was used for the detection of pY321 in DYRK1A. The cross-reaction with pY321 is due to the sequence similarity of the activation loops in HIPK2 and DYRK1A [6].

#### Cell culture and transient transfection

HeLa cells were cultivated in RPMI1640 medium supplemented 10% fetal calf serum at 37 °C and 5% CO_2_. Cells were transfected with plasmid DNA using Fugene HD (Promega, Mannheim, Germany) according to the manufacturer’s instructions.

#### Immunoprecipitation and kinase assay

Transiently transfected HeLa cells were lysed with precooled immunoprecipitation buffer (50 mM Tris–Cl pH 7.5, 150 mM NaCl, 15% glycerol, 2 mM EDTA, 1 mM Na_3_VO_4_) supplemented with the non-denaturing detergent Igepal-CA630 (0.5%) and protease inhibitors (1 mM phenylmethylsulfonyl fluoride and 10 μg/mL each of aprotinin, pepstatin and leupeptin, 1 mL per 10-cm plate) by agitating the plates on ice for 20 min. Lysates were transferred to test tubes, sonicated and centrifuged (5 min, 14.000 rpm, 4 °C). The recombinant GFP-tagged DYRK1A fusion proteins were purified by immunoprecipitation with GFP-Trap_M (ChromoTek, Martinsried, Germany; 1 h at 4 °C in an end-over-end rotator. The antibody-coupled paramagnetic beads were collected using a magnetic rack, washed and equilibrated with kinase buffer (25 mM Hepes pH 7.4, 0.5 mM dithiothreitol, 5 mM MgCl_2_). Samples were assayed for kinase activity with 20 μM DYRKtide and 10 μM [γ–^32^P]ATP (Hartmann Analytics, Braunschweig, Germany) in a total volume of 40 μL for 7 min at 30 °C. Incorporation of ^32^P into DYRKtide was determined in triplicate by the phosphocellulose method. The amount of GFP-DYRK1A protein in the assays was measured by Western blot assays.

#### In vitro translation

We used a bacterial cell-free transcription/translation system (PURExpress, New England Biolabs, Beverley, MA, USA) to investigate the co-translational tyrosine autophosphorylation of DYRK1A. This system is reconstituted from purified components necessary for *E. coli* translation, which excludes the presence of undefined cofactors, chaperones or other protein kinases that may influence the folding, maturation and tyrosine autophosphorylation of the catalytic domain of DYRK1A [2]. Expression was driven by pET-ST2-DYRK1A_28–499_, which encodes a DYRK1A deletion construct fused to an N-terminal Strep-tag 2 sequence [6]. Reactions were incubated in a volume of 10 μL with 10 ng/μL plasmid DNA at 37 °C for 90 min. Autophosphorylation of Y321 in DYRK1A was assessed by immunoblot blot with rabbit anti phospho-HIPK2 (Tyr361) antibody. Band intensities were normalized to total protein levels as detected by Strep-Tactin conjugated to horse radish peroxidase (IBA Life Sciences, Göttingen, Germany).

#### Statistics

The One sample *t* test was used to test the hypothesis that the parameter of interest was different between wild type and mutant DYRK1A (GraphPad PRISM, Graphpad Software Inc.).

## Limitations

- The experiments with DYRK1A-D138P and K150C provide no information whether D138 and K150 are required for optimal in vitro-autophosphorylation of DYRK1A or whether P138 and C150 exert negative effects.
- The effects of the DYRK1A mutations in the in vitro-translation experiment cannot be directly compared with those of DYRK1B, since DYRK1B is not capable of tyrosine autophosphorylation under these conditions.
- The present experiments focus solely on the autophosphorylation of Y321 as a marker for maturation. It would also be of interest to investigate the autophosphorylation of S97, which is an important later step in the folding process [13].
- Additional work will be necessary to reveal whether the observed effects of the substitutions in cell-free translation translate into impaired protein function under conditions of cellular stress.

## Additional files

**Additional file 1.** Documentation of the pET-ST2-DYRK1A_28–499_ expression vector used in Fig. 3.

**Additional file 2.** Assay results underlying the column diagrams in table form.

## Abbreviations

DH box: DYRK homology box
GFP: green fluorescent protein

## References

[1] Lochhead PA, Sibbet G, Morrice N, Cleghon V (2005). Activation-loop autophosphorylation is mediated by a novel transitional intermediate form of DYRKs. Cell.

[2] Walte A, Rüben K, Birner-Gruenberger R, Preisinger C, Bamberg-Lemper S, Hilz N, Bracher F, Becker W (2013). Mechanism of dual specificity kinase activity of DYRK1A. FEBS J.

[3] Becker W, Joost HG (1999). Structural and functional characteristics of Dyrk, a novel subfamily of protein kinases with dual specificity. Prog Nucleic Acid Res Mol Biol.

[4] Kinstrie R, Lochhead PA, Sibbet G, Morrice N, Cleghon V (2006). dDYRK2 and Minibrain interact with the chromatin remodelling factors SNR1 and TRX. Biochem J..

[5] Keramati AR, Fathzadeh M, Go GW, Singh R, Choi M, Faramarzi S (2014). A form of the metabolic syndrome associated with mutations in DYRK1B. N Engl J Med.

[6] Abu Jhaisha S, Widowati EW, Kii I, Sonamoto R, Knapp S, Papadopoulos C, Becker W (2017). DYRK1B mutations associated with metabolic syndrome impair the chaperone-dependent maturation of the kinase domain. Sci Rep..

[7] Himpel S, Panzer P, Eirmbter K, Czajkowska H, Sayed M, Packman LC (2001). Identification of the autophosphorylation sites and characterization of their effects in the protein kinase DYRK1A. Biochem J..

[8] Soundararajan M, Roos AK, Savitsky P, Filippakopoulos P, Kettenbach AN, Olsen JV (2013). Structures of Down syndrome kinases, DYRKs, reveal mechanisms of kinase activation and substrate recognition. Structure..

[9] Widowati EW, Ernst S, Hausmann R, Müller-Newen G, Becker W (2018). Functional characterization of DYRK1A missense variants associated with a syndromic form of intellectual deficiency and autism. Open Biol..

[10] Sonamoto R, Kii I, Koike Y, Sumida Y, Kato-Sumida T, Okuno Y, Hosoya T, Hagiwara M (2015). Identification of a DYRK1A inhibitor that induces degradation of the target kinase using co-chaperone CDC37 fused with luciferase nanoKAZ. Sci Rep..

[11] Kinstrie R, Luebbering N, Miranda-Saavedra D, Sibbet G, Han J, Lochhead PA, Cleghon V (2010). Characterization of a domain that transiently converts class 2 DYRKs into intramolecular tyrosine kinases. Sci Signal..

[12] Becker W, Weber Y, Wetzel K, Eirmbter K, Tejedor FJ, Joost HG (1998). Sequence characteristics, subcellular localization, and substrate specificity of DYRK-related kinases, novel family of dual specificity protein kinases. J Biol Chem.

[13] Kii I, Sumida Y, Goto T, Sonamoto R, Okuno Y, Yoshida S (2016). Selective inhibition of the kinase DYRK1A by targeting its folding process. Nat Commun..

