# Supplementary material for: Mutational analysis of two residues in the DYRK homology box of the protein kinase DYRK1A
Source: BMC Res Notes. 2018 May 15;11:297. doi: 10.1186/s13104-018-3416-4 (PMC5952693; doi:10.1186/s13104-018-3416-4)
Supplement: Supplementary file 1 — Additional file 1. Documentation of the pET-ST2-DYRK1A28–499 expression vector used in Fig. 3. [file 13104_2018_3416_MOESM1_ESM.pdf]

Additional file 1

Expression plasmid for bacterial expression of DYRK1A$_{28\text{-}499}$

The cDNA sequence encoding amino acids 28-499 of rat DYRK1A (Reference sequence acc. NM_012791.3) was inserted between the *Nhe*I and *Eco*RI sites of the expression plasmid pET-ST2 (Walte et al. 2013) to create an N-terminal fusion with the Strep-tag 2 sequence.

```
   StrepTag2
MAWSHPQFEK NMSHMASLQM AAQMPHSHQY SDRRQPNISD QQVSALSYSD QIQQPLTNQV
MPDIVMLQRR MPQTFRDPAT APLRKLSVDL IKTYKHINEV YYAKKKRRHQ QGQGDDSSHK
KERKVYNDGY DDDNYDYIVK NGEKWMDRYE IDSLIGKGSF GQVVKAYDRV EQEWVAIKII
KNKKAFLNQA QIEVRLLELM NKHDTEMKYY IVHLKRHFMF RNHLCLVFEM LSYNLYDLLR
NTNFRGVSLN LTRKFAQQMC TALLFLATPE LSIIHCDLKP ENILLCNPKR SAIKIVDFGS
SCQLGQRIYQ YIQSRFYRSP EVLLGMPYDL AIDMWSLGCI LVEMHTGEPL FSGANEVDQM
NKIVEVLGIP PAHILDQAPK ARKFFEKLPD GTWSLKKTKD GKREYKPPGT RKLHNILGVE
TGGPGGRRAG ESGHTVADYL KFKDLILRML DYDPKTRIQP YYALQHSFFK KTADEGTNTS
NSVSTSPAM
```

Amino acid sequence of the ST2-DYRK1A$_{28\text{-}499}$ fusion protein

The N-terminal StrepTag2 sequence and the DH box are boxed. The catalytic domain is underlined. The amino acids targeted by the mutant constructs in Fig. 3 are marked by blue circles and the autophosphorylated Y321 by a red circle.

Calculated molecular mass: 57 kD.
